# School tobacco policies and social inequalities in adolescent smoking: Nora Mélard

**DOI:** 10.1093/eurpub/ckac129.162

**Published:** 2022-10-25

**Authors:** N Mélard, V Lorant

**Affiliations:** Institute of Health and Society, UCLouvain, Brussels, Belgium

## Abstract

**Background:**

Adolescents of lower socio-economic status initiate smoking earlier and smoke more frequently than those of higher socio-economic status. Tobacco control policies, such as school tobacco policies, aim to reduce adolescent smoking, but their implementation has been found to vary greatly from one school to another. Such differences in the implementation might therefore contribute to social inequalities in smoking. This study examines whether school tobacco policies are implemented where they are most needed, and how this implementation according to needs has changed over time.

**Methods:**

Student (n = 18,805) and staff surveys (n = 438) were conducted in 2013 and 2016 in 38 schools from six European cities in six countries. School tobacco policies were measured as a 10-point score taking into account their multidimensionality, and the perceptions of both students and staff. We used concentration curves and indices to measure the inequality in the implementation of these policies depending on the smoking prevalence and on adolescents’ socio-economic status.

**Results:**

A concentration curve below the perfect equity line indicated a concentration of school tobacco policies where smoking prevalence was lower. Moreover, this inequality was larger in 2016 compared to 2013 (concentration indices of .038 in 2013 and .041 in 2016). On the contrary, a concentration curve overlaying the perfect equity line indicated no inequality in the implementation of these policies depending on adolescents’ socio-economic status (concentration indices of .016 in 2013 and -.013 in 2016).

**Conclusions:**

School tobacco policies have been developed to reduce adolescent smoking. They, however, seem to be less implemented in schools where they are most needed. This confirms that smoking prevention is still driven by the inverse prevention law. Next to evaluating the impact of such policies on smoking outcomes, research should also focus on their contribution to social inequalities in adolescent smoking.

**Key messages:**

• School tobacco policies, developed to reduce adolescent smoking, might contribute to social inequalities in smoking.

• School tobacco policies are less implemented where they are most needed.

